# Toward Successful International Pooling of Breast Implant Registry Data: The Role of Dataset Uniformity

**DOI:** 10.1093/asjof/ojag070

**Published:** 2026-04-25

**Authors:** Uwe Von Fritschen, Iris Moes, Susannah Ahern, Patrick Garduce, Puck Melse, Marco Ventimiglia, Hinne Rakhorst, Marc A M Mureau, Martin Halle, Antonella Campanale, Birgit Stark, Benoit Couturaud, Shiv Chopra, Katie Sommers, Alexandra-Calina Bumbuc

## Abstract

**Background:**

Despite widespread clinical use, the long-term performance of breast implants remains debated. Current evidence is limited by small sample sizes and inconsistent methodologies. National breast device registries provide valuable real-world data, yet their impact is constrained by heterogeneity in variable definitions, data structures, and analytical approaches. The International Collaboration of Breast Registry Activities (ICOBRA) initiative established a harmonized dataset utilized by several countries.

**Objectives:**

Evaluate the implementation of the dataset of the ICOBRA across national registries, identify inconsistencies in data definitions and structures, and develop an updated international dataset supporting standardized, federated analyses.

**Methods:**

Datasets from 5 national breast implant registries utilizing the harmonized dataset were systematically reviewed. Variations in data points and definitions were identified and discussed through a series of in-person and online consensus meetings. Structured voting was used to finalize revisions and establish the updated international core dataset.

**Results:**

Key discrepancies were identified in the classification of surgical indications, inclusion of implant types, and variable definitions across registries. As a result, more than half of the original core variables required revision, reclassification, or removal to enable multinational analyses. A federated analysis model was proposed to accommodate privacy regulations and registry diversity while supporting standardized, risk-adjusted analyses.

**Conclusions:**

The updated international breast implant registry dataset represents a crucial step toward integrated global surveillance. Together with standardized statistical methods within a federated data model, it enables robust pooled analyses without requiring uniform registry structures. Countries are encouraged to adopt the revised dataset and contribute to a collaborative framework that enhances patient safety through structured, large-scale data collection and analysis.

**Level of Evidence: 5 (Therapeutic):**

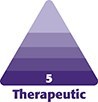

Despite decades of clinical use and extensive literature, breast implants remain a topic of both clinical interest and public debate. One of the most persistent challenges in the field is constraints associated with the collection of high-quality, longitudinal, and standardized data to monitor long-term device performance and patient outcomes. Previous clinical studies are often limited in scale and duration, resulting in fragmented evidence that is insufficient to detect rare but clinically significant complications. To address this gap, the International Collaboration of Breast Registry Activities (ICOBRA), a suborganization of the International Confederation of Plastic Surgery Societies, was established in 2013 to standardize core datasets and definitions across national breast implant registries, thereby enabling meaningful cross-country comparisons and international data pooling. For over a decade, ICOBRA has supported improved postmarket surveillance of breast implants, facilitated signal detection for implant-related adverse events, informed regulatory policy, and enhanced clinical decision making.

In an initial step toward harmonization, ICOBRA published its first standardized core dataset, defining a set of essential variables and their corresponding data definitions in 2020.^1^ This framework provided a foundation for international comparability and enabled a structured approach to registry implementation in multiple countries.^2,3^ Since then, national-level registries have made significant progress in collecting data in alignment with this core structure, allowing for preliminary insights into trends, complication rates, and surgical practices.

However, meaningful integration of data across national registries has remained challenging. Early attempts at data pooling exposed significant issues, including differences in variable definitions, inconsistent classification of surgical indications, structural variations in registry designs, and heterogeneous levels of data completeness. These inconsistencies not only hinder joint analyses but also limit the comparability of national annual reports.^4^ Given the low incidence of serious adverse events associated with breast implants, national datasets alone often lack the statistical power needed to identify safety signals within a reasonable timeframe. This underscores the importance of international data aggregation. Comparable harmonization initiatives among international arthroplasty registries have yielded significant benefits for patients, clinical practice, and the medical device industry, including research and development efforts, as valuable role models.^5,6^

Two proof-of-concept studies for breast implants were conducted to explore the feasibility of pooling data across ICOBRA-participating registries, considering the specifics of the available national data as well as existing legal frameworks. The first study successfully combined real-world datasets from Australia, the Netherlands, Sweden, and the United States in 2021.^7^ Although the analysis demonstrated that technical integration is feasible and allowed for the identification of general trends, national variations, differences in implant preferences, and overall revision rates, it also revealed inconsistencies despite previous harmonization efforts. Key limitations included differences in data structure, variable definitions, exclusion of tissue expanders, incomplete information on legacy devices, and inconsistent grouping of subcategories. As a result, more than half of the initially collected procedures had to be excluded because of lack of compatibility with the standardized definitions. The second study published in 2023 focused on data from the Australian and Dutch registries from 2016 to 2021, which exhibited greater alignment in terms of dataset structure and variable definitions. This targeted approach resulted in fewer exclusions and more consistent analyses, enabling the assessment of real-world cumulative revision incidence rates of breast implants across different implant types.^8^

These 2 studies underscore the potential of combining large datasets to support robust statistical analyses that would be unfeasible using national-level data alone. By pooling information, such evaluations significantly surpass the explanatory power of individual studies and underscore the vital importance of participating in implant registries. However, both studies also illustrated that even subtle variations in registry design and implementation can have a significant impact on data utility and analysis feasibility. Additionally, they brought attention to the legal and ethical challenges posed by varying data protection regulations, which complicates data sharing across jurisdictions.

These findings led to a recognition that a revised and more universally applicable ICOBRA dataset was required, accommodating national differences in registry design while maintaining a robust core suitable for pooled analysis. This study aimed to evaluate the current implementation of the ICOBRA dataset across national breast implant registries and update it through a structured, consensus-based process. The objectives were to identify inconsistencies or barriers to harmonization, revise ambiguous variables, and ensure comparability and feasibility for risk-adjusted analyses across countries.

Accordingly, this manuscript presents an adapted, harmonized ICOBRA dataset that effectively addresses the limitations inherent in the former joined analysis. By implementing the required adjustments, participating registries will be able to jointly analyze a considerably larger volume of data in future investigations.

## METHODS

At the time of this study, 5 national breast device registries were actively participating in ICOBRA harmonization efforts: Australia (Australian Breast Device Registry [ABDR]), the Netherlands (Dutch Breast Implant Registry [DBIR]), Sweden (Bröstimplantatregisrtret [BRIMP]), and more recently Italy (Registro nazionale degli impianti protesici mammari) and Germany (Implantatregister Deutschland), both of which had implemented structured, mandatory breast device registries. Together these countries represented a mixture of opt-out (the Netherlands, Sweden, and Australia) and legally mandated (Germany and Italy) registry models.^9-13^ The current inclusion rate of implants across registries is presented in Table 1; France is included as it will join the group of analyzable registries.

**Table 1.** Total Number of Breast Implants Reported in Participating National Registries (2014-2024)

Patients overall	290.970			
Implants overall	558.454			
			Aesthetic	Reconstruction
Details available	Permanent implants	513.498	338.554	124.944
	Permanent expander	2.527	51	2.476
	Temporary expander	32.819	321	32.498
Overall		548.844	159.918	388.926

Total number of implants included from 2014 to 2024 in the registries of Australia, the Netherlands, Sweden, France, Italy, and Germany. A total of 558,454 implants from 290,970 patients were included. For 548,844 implants, details on implant type and indication could be extracted according to the previous dataset.

### Delphi Analysis of Data Elements

Between January and June 2024, 2 project coordinators developed a registry data dictionary template based on the 2020 ICOBRA core dataset. This template was distributed to the 5 participating national registries. Data elements were categorized into 4 domains: patient-related, device-related, surgery-related, and complication-related variables. Each registry completed the template by listing their currently collected data elements along with corresponding definitions.

The returned templates were systematically reviewed for alignment with the ICOBRA core definitions. Subsequently, a series of individual web-based meetings was conducted with each national registry team to validate the findings and resolve ambiguities. Identified discrepancies were classified as structural deviations (eg, differences in variable format or missing fields), definitional inconsistencies (eg, divergent interpretations of indications or outcomes), and procedural inconsistencies (eg, variations in how surgical procedures or revisions were recorded). Each deviation was assessed for its clinical and statistical relevance, based on insights derived from 2 previous proof-of-concept studies.^7,8^ Variables found to be inconsistently collected were flagged for modification or removal. An in-person consensus workshop was held in September 2024 to align priorities and initiate dataset revision.

Subsequently, a modified Delphi approach was conducted online using Qualtrics XM software (Qualtrics, Provo, UT). A modified Wideband Delphi Technique was selected, as it allows greater interaction and communication among participants than the traditional Delphi method, facilitating interparticipant alignment and more rapid consensus building. The process consisted of 2 structured survey rounds supplemented by videoconferences and a final in-person meeting.

Two registry representatives (ultimately comprising 8 clinicians and 4 data scientists/registry managers) from each participating country independently evaluated each data element and the proposed actions: designation as mandatory or optional, removal, replacement with an alternative variable, or referral for further discussion. Data elements without unanimous agreement were adjudicated using a predefined consensus threshold of 80% approval. After each round, variables that failed to reach consensus were revised and included in the subsequent round. Following completion of the surveys, 2 online consensus meetings were held in March and April 2025 to review each data element in detail and resolve remaining discrepancies (Supplemental Figure 1). Data elements or definitions that could not be fully harmonized across all participating countries or were identified as difficult to integrate because of country-specific structural constraints were deferred, with the aim of integrating them into subsequent update cycles of the affected national registries. The final consensus dataset was then circulated to the clinical leads of each participating country for formal approval.

## RESULTS

In the current assessment, 25 out of the original 51 data points deviated from the agreed core dataset. These discrepancies included variations in the reporting of short-term outcome variables and clinical aspects that lead to unplanned revisions or explantations not related to implant failures, such as implant extrusion because of wound breakdown, hematoma, or infection. In contrast, reasons for revision relevant to the long-term evaluation of specific implants, such as capsular contracture or rupture, were generally well represented.

The implementation analysis revealed 3 principal categories of deficiencies across participating registries. First, heterogeneous aggregation of surgical indications resulted in the conflation of clinically distinct risk profiles, limiting comparability of revision outcomes across countries. Second, inconsistent inclusion of implant types, most notably temporary and permanent tissue expanders, led to structural incompatibilities in defining primary implantation and revision procedures. Third, variation in the definition and reporting of revision events, including implant exchanges, revisions without replacement, and secondary implantation after previous explantation, reduced the interpretability of pooled outcome measures.

As a consequence of these findings, the revised ICOBRA dataset introduced more granular indication categories, removed or redefined data elements that were inconsistently captured, and clarified the definitions of primary implantation, revision, and implant exchange. These changes were designed to maximize cross-registry compatibility while preserving clinically meaningful distinctions required for valid international analyses.

All 51 core data elements were re-evaluated, resulting in revisions to 29 variables (see Supplemental Figure 1). Definitions were modified for 7 variables (date of birth, gender, primary/secondary intervention, autoimmune syndrome induced by adjuvants [ASIA], flap cover, fat grafting, and preoperative antibiotics), 8 new data points were added (type of intervention: first insertion of a breast device/implant exchange/implant insertion after previous explantation; breast implant associated—B-cell lymphoma/squamous cell carcinoma; skin necrosis/exposed implant; acellular dermal matrix [ADM]/Mesh-related complication; entirely submuscular placement; removal ADM/Mesh; device manufacturer; rinse of pocket without active component), and 11 variables were removed because of inconsistent collection or limited analytical value across registries (type of intervention primary/secondary/revision, skin scarring problem, flap problem, silicone extravasation, neo-pocket formation, nipple absent, marker/medical record of explanted device if known, volume of tissue expander, and volume of removed implant). Free-text options were removed for implant position and rinse of pocket. Three variables (hematoma, asymmetry, and date of insertion of removed implants), which narrowly missed the predefined 80% consensus threshold, were reclassified from mandatory to optional. Conversely, 3 variables (sex; ASIA/breast implant illness [BII]/systemic symptoms associated with breast implants [SSBI]; and breast cancer, newly diagnosed or recurrence) were upgraded from optional to mandatory based on emerging analytical relevance and practical feasibility identified during previous joint analyses. These adaptations were intended to ensure the feasibility of international pooled analyses while minimizing mandatory data collection and addressing data scarcity, thereby preserving a stable and comparable international core dataset. Deviations, the resulting adaptations, and justification are presented in Table 2; corresponding changes in data definitions are detailed in Supplemental Table 1.

**Table 2.** Changes of the Previous Dataset

List of previous data points	Changed into the following new data points	Notes on changes
Patient related		
Date of birth/age of patient	Age of patient	“Date of birth” caused privacy issues in several countries
Gender	Sex	“Biological sex at birth” improves risk stratification; previously optional
Surgery related		
Type of intervention Primary Secondary Revision Explantation only Repositioning of an existing implant	First insertion of a breast device Implant exchange Implant insertion after previous explantation Explantation only Repositioning of an existing implant	Data point revised because of collection discrepancies, and in part ambiguous definition point (e) remains optional as it is inconsistently gathered across countries
Reasons for revision		
ASIA	ASIA/BII/SSBI	Previously optional in the Global Dataset; now mandatory as BII/SSBI has emerged as a common revision indication
	BIA-BCL/SCC	Added, other implant-related pathologies previously not collected
Newly diagnosed breast cancer	Breast cancer, newly diagnosed, or recurrence	Previously optional
Hematoma		Previously mandatory; now optional as not implant related
	Skin necrosis/exposed implant	Added. Broader definition than “skin scarring problem”
Skin scarring problem		Removed. Substituted by “skin necrosis/exposed implant”
Flap problem		Removed since not implant related
Silicone extravasation		Removed since redundant
Asymmetry		Turned into an optional data point
	ADM/Mesh-related complication	Added. Potential new source of complications
Details of procedure		
Implant position plane Subglandular Subfascial Subflap Subcutaneous Dual plane Others (please specify)	Implant position plane Subglandular Subfascial Subflap Subcutaneuous Dual plane Entirely submuscular	“Entirely submuscular” was missing as an implantation plane Free-text option removed
Neo-pocket formation		Removed. Not necessary for assessing implant performance Redundant to “Partial capsulectomy”
Flap cover	Concurrent flap cover	Previous flaps are not relevant for current risk assessment
Fat grafting	Concurrent fat grafting	Previous fat grafting not relevant for current assessment
Nipple absent		Not relevant to evaluate implant performance and safety
Intraoperative measures		
Rinse of pocket Antibiotics Antiseptics Others (please specify)	Rinse of pocket Antibiotic solution Antiseptic solution Without active component	Adapted for clinical relevance and usage; some components not permitted in all countries Free-text option removed
Preoperative antibiotics	Preoperative antibiotics	Definition adapted to reflect clinical relevance and usage
	Removal ADM/Mesh	Newly included because of emerging clinical relevance
Implant information		
Date of insertion of removed implants		Made optional, since the data collection is unreliable
Device details of explanted device Texture Fill Shape	Device details of explanted device Texture Fill Shape Manufacturer	Subpoint d) added to replace “Marker/medical record of explanted device if known”
Marker/medical record of explanted device if known		Removed. This data point was redundant and collection unreliable
Volume of tissue expander		Removed. Not relevant for implant performance
Volume of removed implant		Removed. This data point was redundant

Modifications introduced in this ICOBRA update, encompassing adjustments to the relevance for data collection (mandatory or optional) and the removal of specific items. Corresponding changes in data definitions are detailed in Supplemental Table 1. ADMs, acellular dermal matrixes; BIA-BCL/SCC, breast implant associated–B-cell lymphoma/squamous cell carcinoma; BII, breast implant illness; SSBI, systemic symptoms associated with breast implants.

Patient-identifying data were collected by all registries, with variations based on national requirements and legal conditions. Although all registries aimed to fulfill the track-and-trace function to the extent possible, this was considered a national concern and not included in the harmonization.

Two of the 5 registries, including Sweden (BRIMP) and Australia (ABDR), were established with a strong emphasis on postmarket device surveillance, whereas the remaining registries incorporated broader clinical quality objectives. These differing primary objectives influenced both variable selection and data granularity.

Conceptual inconsistencies created significant challenges, particularly regarding the heterogeneous grouping of subcategories. As a direct consequence, substantial proportions of otherwise eligible procedures had to be excluded from previous pooled analyses, underscoring the need to refine indication categories to restore analytical validity and cross-country comparability. For example, “breast reconstruction” was variably used to encompass oncologic, congenital, gender-affirming, and benign indications—each with distinct risk profiles that, if aggregated, distort cross-country comparisons. This heterogeneity also affected the reported cumulative all-cause revision incidence and limited the ability to assess specific implant-related factors associated with each indication. Consequently, several indication categories required refinement to restore comparability.

Some variables or implant types, such as temporary tissue expanders, were excluded from specific national datasets (eg, Sweden), complicating the definition of “primary procedure” as well as “implant exchange.” The high prevalence of permanent tissue expanders in Sweden also complicated the analysis, because these are relatively frequently exchanged for permanent implants, but without any given timeline.^14^ Additionally, some mandatory items, such as BMI or age at surgery, were captured in different formats. Some countries recorded height and weight to derive BMI; similarly, some used age or the exact dates of birth. Because it is possible to calculate a common data point from the available primary inputs, these discrepancies were not addressed.

As a result of the Delphi process, multiple variables were revised, merged, or removed. The final version of the updated mandatory dataset is presented in Table 3. Optional variables and unresolved discrepancies are summarized in Tables 4 and 5, respectively. A full list of all data elements and definitions is available in Supplemental Table 1.

**Table 3.** New List of the Mandatory Data Points.

Patient related	1. Age of patient
	2. Sex
	3. Height
	4. Weight
Case related	7. Laterality
Previous treatment	8. Previous radiotherapy
Surgery related	9. Type of intervention First insertion of a breast device Implant exchange Implant insertion after previous explantation Explantation only
	10. Timing of reconstruction Immediate Delayed
	11. Indications for surgery Cosmetic augmentation Reconstruction after risk reducing mastectomy Reconstruction, benign Reconstruction after mastectomy for cancer
	12. Reasons for revision/explantation Patient preference Asymptomatic Complication
Reasons for revision	13. ASIA/BII/SSBI
	14. BIA-ALCL suspected
	15. BIA-ALCL confirmed
	16. BIA-BCL/SCC
	17. Breast cancer, newly diagnosed or recurrence
	18. Breast pain
	20. Device malposition/rotation
	22. Skin necrosis/exposed implant
	23. Device rupture
	24. Infection leading to explanation
	25. Capsular contracture
	27. ADM/Mesh-related complication
	28. Seroma
Details of procedure	29. Incision site Inframammary Periareolar Axillary Mastectomy scar Others
	30. Implant position plane Subglandular Subfascial Subflap Subcutaneous Dual plane Entirely submuscular
	31. Capsulectomy Partial capsulectomy Complete capsulectomy
	32. Concurrent mastopexy
	33. Concurrent flap cover
	34. Concurrent fat grafting
	35. Nipple sparing mastectomy
Intraoperative measures	36. Rinse of pocket Antibiotic solution Antiseptic solution Without active component
	37. Preoperative antibiotics
	38. Postoperative antibiotics
	39. Glove change before insertion
	40. Drain
	41. ADM/Mesh used
	42. Device details of the ADM/Mesh used
Implant information	45. Device manufacturer
	46. Device lot number
	47. Catalogue reference number
	48. Device serial number
	49. Device shape
	50. Texture
	51. Fill
	52. Volume of implant
	54. Device details of explanted device Texture Fill Shape Manufacturer

Presents the new harmonized ICOBRA dataset of mandatory collected data points. ADMs, acellular dermal matrixes; AISA, autoimmune syndrome induced by adjuvants; BIA-BCL/SCC, breast implant associated–B-cell lymphoma/squamous cell carcinoma; BII, breast implant illness; SSCI, systemic symptoms associated with breast implants.

**Table 4.** New List of the Optional Data Points

List of the optional data points
Patient related
5. ASA classification
6. Smoking
Surgery related
9. Type of intervention (see also Table 2) e) Repositioning of an existing implant
19. Double capsule
21. Hematoma
26. Asymmetry
Intraoperative measures
42. Occlusive nipple shields
43. Removal ADM/Mesh
Implant information
52. Date of insertion of removed implants

Presents the new harmonized ICOBRA dataset of optionally collected data points.

**Table 5.** Remaining Discrepancies in Active Registries Not Yet Aligned

List of the global data points	Changes in the new global data set	Status	Discrepancies in national registries
Patient related			
Gender	Sex	M	Australia collects “Gender” (Default: F/M/Other)
Timing of reconstruction Immediate Delayed	Unchanged during this update	M	This data point is not collected in Sweden
Indications for surgery Cosmetic augmentation Reconstruction after risk reducing mastectomy Reconstruction, benign Reconstruction after mastectomy for cancer	Unchanged during this update	M	Sweden does not explicitly collect cosmetic procedures; they are comprehended under the subgroup “benign”
Reasons for revision			
	BIA-BCL/SCC	M	Sweden: The registry currently does not collect this data point Australia: The registry currently does not collect this data point Italy: The registry currently does not collect this data point
Newly diagnosed breast cancer	Breast cancer, newly diagnosed or recurrence	M	Sweden: The registry currently does not collect this data point
	Skin necrosis/exposed implant	M	Sweden: The registry currently does not collect this data point Australia: The registry currently does not collect this data point Italy: The registry currently does not collect this data point
	ADM/Mesh-related complication	M	Sweden: The registry currently does not collect this data point but can analyze complications related to both as an aggregated variable Australia: The registry currently does not collect this data point The Netherlands: The registry currently does not collect this data point Italy: ADM/mesh usage is recorded, but establishing a causal relationship with postoperative complications remains difficult
Details of procedure			
Implant position plane Subglandular Subfascial Subflap Subcutaneous Dual plane Others (please specify)	Implant position plane Subglandular Subfascial Subflap Subcutaneous Dual-plane Entirely submuscular	M	Sweden: does not specifically register “subcutaneous” as a separate category, only records “subglandular/prepectoral” as a single combined variable Australia: combines “subcutaneous” and “prepectoral” into 1 category (“subcutaneous/prepectoral”)
Concurrent mastopexy		M	Sweden: collects this data point only for primary operations
Intraoperative measures			
Rinse of pocket Antibiotics Antiseptics Others (please specify)	Rinse of pocket Atibiotic solution Antiseptic solution without active component	M	Sweden: The registry only collects antibiotic rinse Australia: The registry collects “antibiotic”/“antiseptic”/“other (please specify)”
Glove change before insertion		M	Sweden: The registry currently does not collect this data point
Drains		M	Sweden: The registry currently does not collect this data point
ADM/Mesh used		M	Sweden: The registry currently does not collect the specific type, but solely ADM/Mesh as a dichotomous variable
Device details of the ADM/Mesh used		M	Sweden: The registry currently does not collect device details
	Removal ADM/Mesh	O	Sweden: The registry currently does not collect this data point The Netherlands: The registry currently does not collect this data point Australia: The registry currently does not collect this data point Italy: The registry currently does not collect this data point
Implant information			
Device lot number		M	Sweden: The registry currently does not collect this data point; only the device serial number is collected
Catalog reference number		M	Sweden: The registry currently does not collect this data point

Remaining ICOBRA data points that could not be harmonized across all actively participating countries, along with the reasons for these discrepancies. Status: O = optional, M = Mandatory. ADM, acellular dermal matrix; BIA-BCL/SCC, breast implant associated—B-cell lymphoma/squamous cell carcinoma.

## DISCUSSION

The implementation of the harmonized ICOBRA dataset, alongside the expansion of real-world registry data, has advanced evidence-based evaluation of breast implants. Although adopting comparable datasets represents an essential first step, expanding data through international collaboration is crucial for detecting rare events and emerging patterns. These efforts have already yielded valuable insights but have also revealed persistent challenges, including differences in registry objectives, variable definitions, risk stratification methods, and gaps in capturing certain implant types or procedures.

Established in 2013, ICOBRA aimed to generate comparable data for early detection of implant failures. This study builds on that goal by addressing deviations from the core dataset and inconsistencies limiting analytical validity. Two preceding multicountry studies successfully utilized large registry-based ICOBRA samples to overcome the limitations of isolated datasets and enabled evidence-based assessment of revision rates across implant groups.^7,8^ However, despite alignment under the ICOBRA harmonized dataset, participating registries continued to exhibit systematic heterogeneity in variable definitions and data completeness. This heterogeneity, combined with inconsistent classification of surgical indications, nearly halved the analyzable dataset and necessitated a substantial narrowing of research questions and assessed indications, underscoring the need for renewed harmonization efforts.

In addition, despite formal alignment and standardization, outcomes could only be presented as crude, unadjusted data, thereby limiting the internal validity of direct cross-country comparisons unless appropriately modeled and transparently contextualized. The inclusion of newly established registries, still in early phases of data capture and limited representativeness, not yet fully reflecting national practice, may require specific statistical adaptations in future analyses, including phased or staged approaches that account for registry inclusion rates at defined time points.

### Dataset Refinement and Variable Classification

Evolving and continuously refined variable definitions impact longitudinal comparisons, potentially affecting cohort stability. Efforts were made to minimize the impact, and in the present revision, only a limited number of variables are affected. Most modifications involve reclassifying variables as mandatory or optional. Likewise, the inclusion of subcategories within umbrella terms used by some countries resulted in the exclusion of affected datasets from previous specific analyses. We anticipate that the updated dataset will enable more granular and standardized data capture, facilitating inclusion in joint analyses.

The terminology used to describe patient-reported systemic symptoms varies across national registries. Although some registries have historically used the term ASIA, others refer to BII or SSBI. In the revised dataset, these terms were harmonized into a single data element capturing patient-reported systemic symptoms, without implying a specific underlying pathophysiological mechanism. This approach reflects the absence of a recognized ICD-10 (International Statistical Classification of Diseases and Related Health Problems) diagnosis and the ongoing scientific debate regarding causality, while ensuring consistent and comparable capture of patient-reported outcomes relevant to international surveillance efforts.

The only modification of substantial methodological relevance was the more precise definition of “primary implantation,” because the original definition had led to misunderstandings in clinical practice. Other refinements, such as clarifying that flap coverage or fat grafting refers to a concurrent procedure, are of comparatively minor relevance. Consequently, historical analyses can continue to rely on consistent datasets, whereas the refined definitions will allow the inclusion of previously ambiguous data. Prospective and retrospective evaluations of individual cohorts, based on inclusion rates and observation periods, will follow established practice.

### Data Protection and Federated Analysis

A critical barrier to cross-national data pooling remains the diversity of legal and regulatory frameworks governing data privacy. The European Union's (EU’s) General Data Protection Regulation imposes strict conditions on the collection, processing, and transfer of personal health data.^15^ Although research exemptions exist, these must be interpreted and authorized within each national context, often involving ethics board approvals and specific institutional agreements.

In the first proof-of-concept study, to address these challenges, a federated learning approach was applied.^7^ Local analyses were conducted separately within each registry using a standardized script-based statistical methodology. Only fully anonymized, aggregate-level outputs were shared for central meta-analysis. This approach ensured compliance with national regulations and preserved patient confidentiality while enabling comparative evaluation.

In the second study, anonymized Dutch registry data were securely imported into a controlled e-research environment in Australia.^8^ Access was granted under a guest researcher agreement that imposed strict data access limitations. All exports were screened and approved to ensure no identifying data left the secure environment. The analysis was conducted exclusively by a designated researcher, without direct data access by the host country's registry team.

These experiences have demonstrated that the structure of future international analyses must be substantially simplified in order to be feasible. In doing so, the statistical challenges arising from the aggregation of data across registries with differing structures and levels of maturity must also be taken into account.

### Statistical Challenges

An ongoing challenge in evaluating implant outcomes is the definition and accuracy of denominators, that is, the total number of procedures performed. However, across registries, definitions of completeness vary. Some rely on the proportion of institutions reporting data, whereas others estimate based on manufacturer sales data or a combination of other information. Both approaches have limitations. Institutional participation rates may be inflated by including low-volume centers, whereas sales data can be distorted by stockpiling or unrecorded usage. Capture rates vary between registries too. Mandatory systems generally perform best, with rates exceeding 90%. Opt-out systems represent a significant improvement over opt-in approaches, but they often still fall short, particularly in revision or aesthetic procedures, where verification is not possible. Explantations without replacement and undocumented revisions remain particularly difficult to quantify. As such, current revision rates reported in national registries likely represent a conservative estimate, capturing only a subset of actual adverse outcomes.

Furthermore, the presence of legacy procedures, for example, implants inserted before registry implementation or without complete procedure history, limits the ability to apply time-to-event analyses. In the Australian registry, these cases account for 31% of reconstructive and 23% of cosmetic procedures.^16^ Although such data can support descriptive statistical analysis, they do not support rigorous survival or risk-adjusted modeling. Meaningful analysis will ultimately require the inclusion of implants with consistently and comprehensively recorded data. However, as registries mature over time, the proportion of legacy procedures with incomplete history data will naturally decline. In the interim, a significant portion of the available data can still be utilized in survival analyses.

A more differentiated assessment that includes specific implant specifications presents an additional statistical challenge because of the high degree of heterogeneity among implant products. Although the number of manufacturers is relatively small, the range of specific product types, surface textures, and design modifications is vast. Because the incidence of anomalies leading to revision surgeries, particularly in aesthetic procedures, is so low, drawing a statistically significant conclusion would require a substantial number of devices for each combination of manufacturer and implant type.

To mitigate this limitation, implants were grouped into 4 categories based on their general characteristics. This aggregation has already provided valuable insights, such as overall rupture rates for specific implant groups and the incidence of capsular contracture.^8,17^ It also enabled indication-specific analysis of revision rates and underlying causes across these broader product categories.

International differences in data protection regulations hinder the pooling of complete datasets at a scale required for structured and continuous analyses. Consequently, the use of a federated analytical approach was adopted for future evaluations. Although this strategy cannot fully resolve all statistical challenges, such as structural heterogeneity between registries (eg, differences in maturity, capture rates, and follow-up depth), the harmonization of ICOBRA data elements and definitions provides a robust and well-suited foundation for this approach.

As a future perspective, sophisticated statistical models, including machine learning, might be a solution. Australia is presently exploring possible areas of application. Until this is achieved, the evaluation will remain limited to aggregated implant groups, converted nationally into a common data model, with analyses applied jointly where possible or through meta-analysis.^8^

Additionally, focusing on clearly defined key research questions will reduce the need for extensive variable adaptation and facilitate more straightforward statistical analyses. Potential topics include short-term reasons for revision surgeries, such as underperforming devices, the impact of meshes and ADMs on clinical outcomes, and device-related factors associated with malignancies or ASIA/BII/SSBI. These topics are scheduled for discussion at our upcoming in-person meeting.

### Challenges of Registry Harmonization

As part of the evaluation process, it became clear that national registries cannot be fully harmonized to collect data in a completely uniform manner. Although united under the ICOBRA cooperative, they operate independently with differing data collection methods, providers, and regulatory requirements, creating challenges for consistent data integration. To support more targeted analyses and reduce integration effort, comprehensive adjustments and reharmonization of national datasets were undertaken. Building on the limitations identified in the previous studies, a multilayered approach aligned current national data structures with standardized definitions.

To facilitate pooled analyses while addressing legal and regulatory barriers to data sharing, a federated data model was adopted. Registries continue to collect data according to their national protocols but only extract data in a mutually compatible format for joint aggregated analyses. This approach established a pathway that respects national particularities and data protection requirements while enabling data integration.

Inconsistently aggregated risk profiles present a particular challenge. Conflating subcategories, or grouping multiple potential risk profiles within single data points, leads to ambiguous classification into shared risk groups. For example, the indication “benign” lacks clinical specificity (Table 5), complicating data integration and limiting the evaluation of specific outcomes, including proper adjustment for confounders. To address this, the dataset was revised to ensure that, regardless of national data collection peculiarities, the required information could be clearly identified, extracted, and made available for joint subgroup analyses. Simultaneously, impractical or insufficiently defined data points were revised or removed.

Not all data points were collected consistently across countries. Although some of these variables were optional or reflected national surveillance priorities, they nonetheless influenced other data elements. For example, temporary tissue expanders were excluded because the Swedish registry does not capture this implant type. This exclusion not only led to the loss of relevant cases but also complicated the identification of primary implantations as defined by the ICOBRA core dataset. Likewise, cases where an implant was removed and replaced during a secondary procedure did not fit neatly into either the “revision of an implant” or primary implantation categories.

Extensive changes to national data collection involve considerable intellectual and financial effort and may encounter resistance. Adapting datasets within active registries is a complex process that cannot be repeated on short notice. It requires coordination with registry operators, regulatory authorities, and software providers. Consequently, a small number of data points still cannot yet be collected consistently across all participating countries; these will be incorporated in the future update cycles. Affected data points are listed in Table 5. Accordingly, this dataset review is part of established registry practice and will necessitate a revision of the ICOBRA dataset every 3 to 5 years.

### Market Variability and Product Harmonization

Variation in implant types available across national markets presents additional challenges. For example, the EU's broader range of implant types meant over 700 unique reference numbers could not be included in all analyses of the previous studies. International efforts are underway to harmonize manufacturer-provided product information through Unique Device Identification (UDI). UDI is already mandatory in the EU and the United States, with phased integration based on risk classes in other countries, including Australia.^1,18,19^

### Study Significance

This study demonstrates that substantial variations in variable definitions and their implementation across registries led to the exclusion of a large proportion of otherwise eligible procedures in 2 previous multinational analyses. By systematically identifying and addressing these limitations through an international consensus process involving 5 participating registries, the revised ICOBRA dataset establishes a feasible and robust foundation for future pooled analyses. Importantly, this work shows that meaningful international harmonization can be achieved without requiring uniform registry structures, thereby enabling broader participation and improving the analytical utility of registry data for the evaluation of rare implant-related outcomes.

## CONCLUSIONS

This manuscript presents an adapted harmonized ICOBRA dataset that addresses key limitations previously restricting joint analyses and demonstrates the feasibility and value of international harmonization of breast device registry data. By enabling harmonized data extraction across 5 countries, the revised dataset demonstrates the potential for future large-scale analyses reflecting diverse clinical practices worldwide.

Through a structured, collaborative process, the ICOBRA core dataset was revised to meet current clinical, regulatory, and analytical needs. Critical discrepancies were resolved, and appropriate methodologies for comparative analysis were established. Although complete uniformity among registries remains unattainable, the updated dataset enables extraction of harmonized data blocks independent of local systems by adhering to standardized definitions and analytical methods.

Although legal and structural challenges persist, the framework established here provides a robust foundation for international cooperation in implant surveillance. Additional countries are encouraged to adopt the dataset and contribute to a shared understanding of breast implant safety and performance.

## Supplementary Material

ojag070_Supplementary_Data
