# Supplementary material for: Toward Successful International Pooling of Breast Implant Registry Data: The Role of Dataset Uniformity
Source: Aesthet Surg J Open Forum. 2026 Apr 25;8:ojag070. doi: 10.1093/asjof/ojag070 (PMC13298867; doi:10.1093/asjof/ojag070)
Supplement: ojag070_Supplementary_Data [file ojag070_supplementary_data.zip › Suppl Table 1.docx]

**Supplemental Tab. 1: Comprehensive list of mandatory and optional data points with definitions**

| **List of the Global Data Points** | **Definition** |
| --- | --- |
| **Patient related** |  |
| 1. Age of Patient | As identified in the medical record |
| 1. Sex | Biological sex at birth |
| 1. Height | A person’s self-reported height, measured in centimeters (or inches) |
| 1. Weight | The weight (body mass) of a person measured in kilograms (or pounds) |
| 1. *ASA-Classification* | *A system used by anesthesiologists to stratify severity of patients’ underlying disease and potential for suffering complications from general anesthesia* |
| 1. *Smoking* | *As identified by patient* |
| **Case Related** |  |
| 1. Laterality | Left or the right breast |
| **Previous Treatment** |  |
| 1. Previous Radiotherapy | Radiotherapy to the breast or chest wall at any time before the current device operation |
| **Surgery Related** |  |
| 9. Type of intervention  a) First insertion of a breast device  b) Implant exchange  c) Implant insertion after previous explantation  d) Explantation only  e*) Repositioning of an existing implant* | Type of intervention to include subpoints   1. The first insertion of a new device (i.e., an implant or expander) in a breast 2. Removal of a device and insertion of a new device during the same surgery 3. Device insertion after an explantation, which did not occur during the same surgery 4. Explantation only 5. *Reposition of an existing device, no new device inserted* |
| 10. Timing of reconstruction  a) Immediate  b) delayed | a) Breast reconstruction carried out at the time of mastectomy  b) Breast reconstruction carried out after mastectomy |
| 1. Indications for surgery 2. Cosmetic augmentation 3. Reconstruction after risk reducing mastectomy 4. Reconstruction, benign 5. Reconstruction after mastectomy for cancer | Reason for surgery   1. A cosmetic procedure for enlarging breasts 2. Surgery to reconstruct breast shape and symmetry in patients with absence of one or both breasts due to risk-reducing surgery for breast cancer. 3. Surgery to restore or create shape and symmetry in patients with loss or absence of all or some breast tissue because of benign breast conditions, congenital deformity, tuberous breasts, or gender reassignment surgery 4. Surgical procedures performed to recreate a breast after one or both breasts are removed as a treatment for breast cancer or premalignancies (e.g. DCIS) |
| 1. Reasons for revision/explantation 2. Patient preference 3. Asymptomatic 4. Complication | The main reason for undertaking revision of a breast implant   1. The choice of the patient, i.e. patient preference to change the size of implant, desire to remove/change implant as determined by the patient 2. Procedure performed because of a device recall, or a planned revision, or asymmetry, or revision caused by a complication on the other breast 3. Any deviation from the normal postoperative course |
| **Reasons for revision** |  |
| 1. ASIA/BII/SSBI | Unspecific symptoms due to breast implants, as recognized by patient and surgeon |
| 1. BIA-ALCL suspected | A current or previous diagnosis (pathology based) of BIA-ALCL, where BIA-  ALCL is a CD30+, ALK−, T-cell–derived lymphoma within the non-Hodgkin  lymphoma group; this data point to include “suspected” |
| 1. BIA-ALCL confirmed | A current or previous diagnosis (pathology based) of BIA-ALCL, where BIA-  ALCL is a CD30+, ALK−, T-cell–derived lymphoma within the non-Hodgkin  lymphoma group; this data point to include “confirmed” |
| 1. BIA-BCL/SCC | Other periprosthetic malignancies and/or capsular malignancies |
| 1. Breast cancer, newly diagnosed or recurrence | A current diagnosis of malignant breast disease, including both first-time (de novo) presentations and recurrences after previously completed treatment. |
| 1. Breast Pain | As noted by the patient |
| 1. *Double Capsule* | *A second thin tissue layer encasing the usually textured implant subsequently*  *leading to permanent separation from the outer capsule* |
| 1. Device malposition/rotation | Any instance in which the implant is outside its intended position |
| 1. *Hematoma* | *A collection of blood outside the blood vessels that can be localized in an*  *organ, space, or tissue* |
| 1. Skin necrosis/exposed implant | A postoperative complication characterized by the partial or complete loss of skin viability overlying a breast implant, potentially leading to exposure of the implant surface. |
| 23. Device rupture | Loss of implant shell integrity |
| 24. Infection leading to explanation | An infection associated with a breast implant in place, which leads to its explantation; usually involves redness, localized pain or tenderness, abscess or persistent serous liquid formation around the implant even with distinct clinical signs it might be culture-negative |
| 25. Capsular Contracture | The shrinkage of the foreign body encapsulation scar tissue that forms  around artificial devices imbedded in body tissues |
| 26. Asymmetry | As determined by the patient and identifiable by the surgeon |
| 27. ADM/Mesh related complication | Any adverse event directly associated with the use of an Acellular Dermal Matrix (ADM) or synthetic mesh in breast surgery. This includes complications that are specifically attributable to the presence or behavior of these materials. |
| 28. Seroma | An abnormal accumulation of serum around the device. |
| **Details of Procedure** |  |
| 29. Incision Site  a) Inframammary  b) Periareolar  c) Axillary  d) Mastectomy scar  e) Others | The site where the incision is placed   1. An incision in, or beneath, the inframammary fold 2. An incision around the areola 3. An incision in the axilla 4. An incision at the site of an existing mastectomy incision 5. Any other incision site |
| 30. Implant position plane  a) Subglandular  b) Subfascial  c) Subflap  d) Subcutaneous  e) Dual-plane  f) Entirely submuscular | The surgical plane in which an implant is inserted; this data point to include subpoints  a) subglandular,  b) subfascial,  c) subflap,  d) subcutaneous  e) dual-plane  f) entirely submuscular |
| 31. Capsulectomy  a) Partial Capsulectomy  b) Complete capsulectomy | Removal of the encapsulating scar tissue surrounding the breast implant   1. Surgical release and/or partial removal of the capsule 2. Complete removal of the capsule including thoracic part of the capsule |
| 32. Concurrent mastopexy | Indicating whether the procedure involves a mastopexy (breast lift) |
| 33. Concurrent flap cover | Any type of concurrent flap used for breast reconstruction that covers ab implantable device or adds volume to the breast mound |
| 34. Concurrent fat grafting | Transfer of aspirated fat to the breast region |
| 35. Nipple sparing mastectomy | Removal of the breast tissue with preservation of the breast skin envelope and  the nipple-areola complex |
| **Intraoperative Measures** |  |
| 36. Rinse of pocket  a) Antibiotics  b) Antiseptics  c) Without active component | Rinse of the surgically created pocket before implant insertion   1. Intraoperative wash of the surgical pocket with an antibiotic solution 2. Intraoperative wash of the surgical pocket with antiseptic solution (Iodine, Lavasept, etc) 3. Intraoperative wash of the surgical pocket with saline solution |
| 37. Preoperative antibiotics | Use of antibiotics provided IV, orally or IM directly before incision / within an hour before incision |
| 38. Postoperative Antibiotics | Use of antibiotics provided IV, orally, or IM within 3 hours of surgery completion |
| 39. Glove change before insertion | Change of gloves immediately before insertion of the implant |
| 40. Drain | Intraoperative insertion of drains |
| 41. ADM/Mesh used | The use of either an “absorbable or nonabsorbable synthetic mesh” or  “acellular dermal matrix,” which are medical devices used in breast implant  surgery where the mesh or matrix to provide a soft-tissue scaffold |
| 42. Device details of the ADM/Mesh used | Device details of the inserted ADM/Mesh used |
| *43. Occlusive nipple shields* | *The use of adhesive film dressing covering the nipple-areola complex to prevent perioperative expression of bacteria from nipple ducts contaminating the operative field* |
| *44. Removal ADM/Mesh* | *Removal of a previously implanted ADM/Mesh* |
| **Implant information** |  |
| 45. Device manufacturer | Name of the manufacturer of the implanted device |
| 46. Device lot number | Lot number of the implanted device |
| 47. Catalogue reference number | Catalogue reference number of the implanted device |
| 48. Device serial number | Serial number of the implanted device |
| 49. Device shape | The shape of the device being inserted into or explanted from the breast; where the shape of the device is either round (implant is shaped like a flattened sphere) or shaped (a contoured shape that recreates the more teardrop outline of a mature breast) |
| 50. Texture | The surface texture of the device being inserted or explanted |
| 51. Fill | The material used to fill the breast implant: saline solution, silicone gel, or  Other |
| 52. Volume of implant | As determined by the manufacturer or measured intraoperatively by weight,  or displacement, or fill volume |
| *53. Date of insertion of removed implants* | *Date the explanted implants were inserted (known or estimated)* |
| 54. Device details of explanted device  a) Texture  b) Fill  c) Shape  d) Manufacturer | Any available details of the implant at the time of explantation. Subpoints include   1. Texture 2. Full 3. Shape 4. Manufacturer |

*Suppl. Table 1 presents the complete updated ICOBRA dataset, with optionally collected data points shown in*italic font*.*
