# Supplementary figures and images for: Toward Successful International Pooling of Breast Implant Registry Data: The Role of Dataset Uniformity
Source: Aesthet Surg J Open Forum. 2026 Apr 25;8:ojag070. doi: 10.1093/asjof/ojag070 (PMC13298867; doi:10.1093/asjof/ojag070)

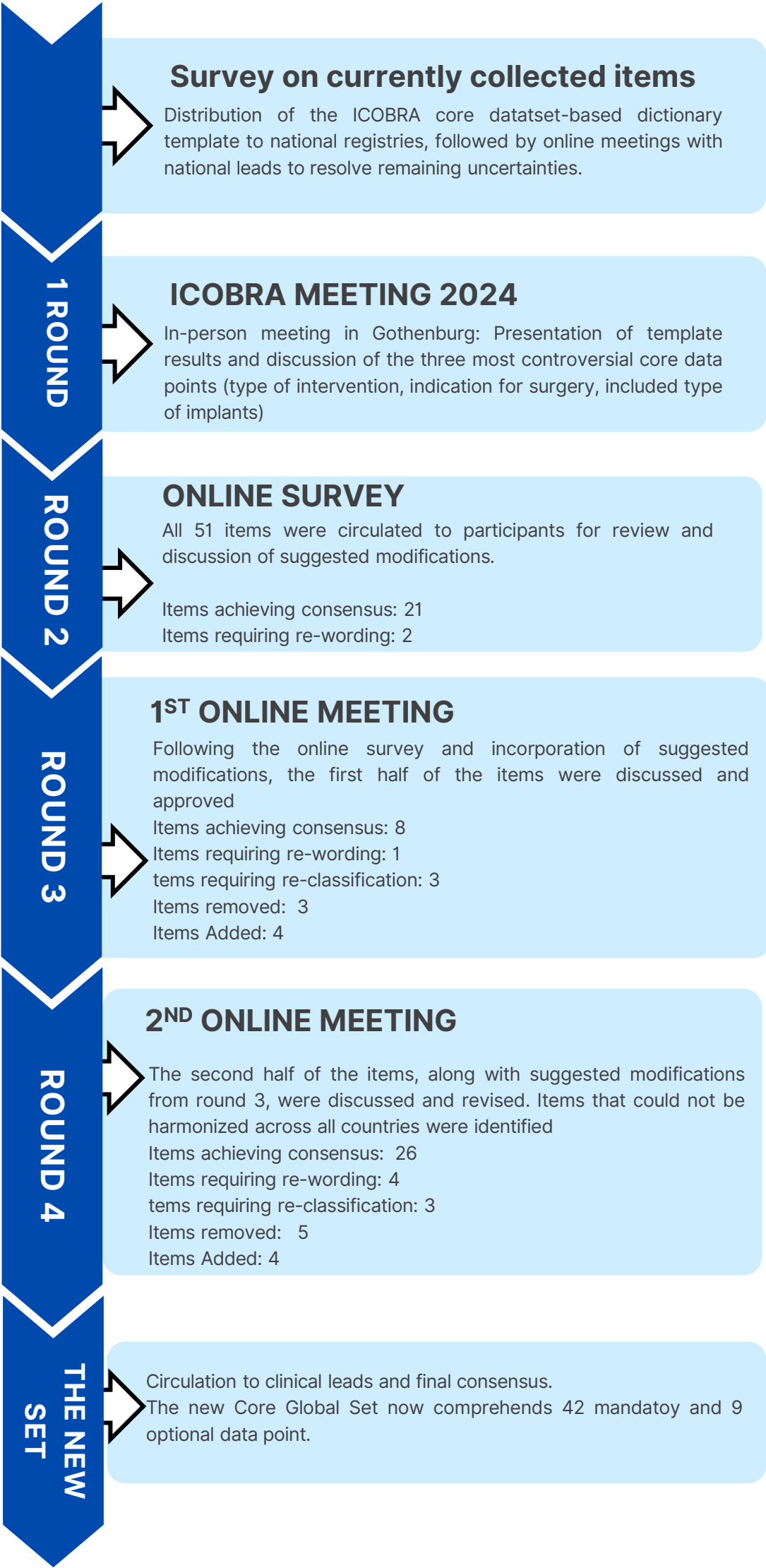

Supplement: ojag070_Supplementary_Data [file ojag070_supplementary_data.zip › Supp. Fig 1_Flow chart.pdf]
